# Meeting Review of the ‘1st Azerbaijan Neuroscience School: Introduction’

**DOI:** 10.1242/bio.061980

**Published:** 2025-05-20

**Authors:** Elkhan Yusifov, Sadig Niftullayev

**Affiliations:** ^1^ Azerbaijan Neuroscience Society, F. Gambarov 76, Baku 1025, Azerbaijan; ^2^ Department of Clinical Chemistry, University Hospital Zurich, Wagistrasse 14, Zurich 8952, Switzerland; ^3^ Department of Psychology, Khazar University, Baku 1009, Azerbaijan; ^4^ Max Planck Institute for Biology of Ageing, University of Cologne, Joseph-Stelzmann-Str. 9b, Cologne 50931, Germany

**Keywords:** Azerbaijan Neuroscience Society, Azerbaijan Neuroscience School, Neuroscience education

## Abstract

The ‘1st Azerbaijan Neuroscience School: Introduction’ program marked a historic milestone as the first neuroscience education initiative in Azerbaijan. This 12-week online program aimed to bridge the gap between Azerbaijani neuroscience enthusiasts and international experts by delivering foundational knowledge, cutting-edge research insights, and critical thinking skills. The program was open to a diverse audience, ranging from high school students to university-level medical and biology students, as well as early-career researchers and faculty members. The virtual format of the program allowed participants to enroll and join in, regardless of geographical location, which fostered scientific exchange, mentorship, and professional networking. The success of this initiative paved the way for the follow-up events: the ‘Azerbaijan Neuroscience School: Advanced’, ‘International Brain Bee Competition’, and the ‘1st Azerbaijan Neuroscience Conference’ in 2024.

## Teaser

**Summary:** This Meeting Review highlights the first neuroscience education initiative in Azerbaijan, showcasing its impact on student training, mentorship, and building a diverse, international neuroscience community.

## Introduction

The field of neuroscience is rapidly evolving, yet access to structured neuroscience education has been limited in Azerbaijan. The ‘1st Azerbaijan Neuroscience School: Introduction’ addressed this gap by providing a comprehensive program aimed at familiarizing students and early-career researchers with current trends, topics and perspectives in neuroscience. Organized by The Azerbaijan Neuroscience Society (ANS) and Neyroelm MMC, and supported by The Company of Biologists, the program connected Azerbaijani learners with experienced scientists from different parts of the globe. The primary goal was not only to teach fundamental knowledge in neuroscience, but also to create a platform for mentorship and collaboration. Due to its online format, the program reached out to a broad audience, including participants from rural regions and those studying abroad. By equipping students with essential research skills and fostering discussions on translational neuroscience, this initiative set the foundation for a growing neuroscience community in Azerbaijan.

### Key themes and discussions

The program was structured to provide an in-depth introduction to key concepts within modern biological sciences and biomedical research, with each session focusing on a distinct topic in neurosciences. The lectures covered topics ranging from scientific literature analysis and experimental design to bioinformatics and clinical neuroscience, as shown in Table 1. Several overarching themes emerged throughout the program:

1. Fundamental biological methodology: Each lecture introduced core principles of a neuroscience-related field, ensuring that participants gained a solid foundation in multiple disciplines.
2. Cutting-edge research: Discussions extended beyond basic principles to showcase ongoing research, providing insights into the latest advancements in the field.
3. Critical thinking and application: A key emphasis was placed on fostering critical thinking, particularly in areas that bridge fundamental neuroscience with clinical applications, such as translational medicine and experimental design.

**Table 1.** Program of the ‘1st Azerbaijan Neuroscience School: Introduction’

Module	Date	Topic	Speaker
**Module 1. Scientific Papers: Proper Use of Scientific Literature**	24 March 2024	Types of scientific papers – research and review articles	Dr. Sadig Niftullayev
**Module 2. Scientific Research: Designing Scientific Experiments**	31 March 2024	Scientific experiments: question, hypothesis, reasoning, and controls	Dr. Sadig Niftullayev
**Module 3. Model Organisms**	7 April 2024	Model organisms	Dr. Artoghrul Alishbayli
**Module 4. Modern Biological Methodology**	14 April 2024	DNA/RNA-related methods	Dr. Madina Guliyeva
	21 April 2024	Biochemical methods	Dr. Sadig Niftullayev
	28 April 2024	Microscopy – light and electron microscopy	Dr. Mahira Safaralizade
	5 May 2024	Cell cultures	Dr. Madina Guliyeva
	12 May 2024	Statistics	Tural Mammadov
	19 May 2024	Bioinformatics	Dr. Elmir Mahammadov
**Module 5. Translational Research**	26 May 2024	Clinical trials	Dr. Rashad Yusifov
**Module 6. Introduction to Neuroscience: Core Concepts**	2 June 2024	Neuroscience: an introduction	Dr. Elkhan Yusifov
**Module 7. Examination**	9 June 2024	Exam preparation	

Some of the most engaging discussions arose in sessions related to the translation of fundamental knowledge into clinical settings, particularly in translational medicine and experimental design. These topics sparked in-depth conversations on how theoretical principles could be applied to real-world medical challenges.

### Speaker highlights

The program featured a distinguished lineup of Azerbaijani early-career researchers, including PhD candidates and postdoctoral researchers working in five different countries, each bringing expertise from leading research institutions. These scientists represent the first generation of neuroscience experts from Azerbaijan. The lectures followed a logical progression, covering core aspects of neuroscience in a structured manner.

The program commenced with Dr. Sadig Niftullayev's lecture (Max Planck Institute for Biology of Aging, Germany), who introduced participants to the fundamentals of scientific methodology and experimental design. His expertise in autophagy research provided a strong foundation for understanding the importance of rigorous experimental approaches. Advancing into methodology, Dr. Madina Guliyeva (University College London, UK) provided in-depth insights into DNA/RNA-related techniques, essential for modern neuroscience research. Dr. Mahira Safaralizade (Freiburg University, Germany) expanded on this by discussing microscopy techniques, emphasizing their role in visualizing cellular structures and processes.

Dr. Elkhan Yusifov (University Hospital Zurich, Switzerland), a postdoctoral researcher in clinical chemistry and the co-founder of the Azerbaijan Neuroscience Society, then provided a comprehensive introductory overview of history and modern trends in neuroscience, discussing relevant fundamental basic science, translational and clinical concepts, which are not otherwise included in the traditional curricula at Azerbaijani institutions. Dr. Yusifov also introduced the students to his area of expertise: the role of primary cilia, as a dynamic signaling hub, for the development of the central and peripheral nervous systems (Yusifov et al., 2021, 2025; Bangs and Anderson, 2017; Baker and Beales, 2009). The lack of cilia leads to a group of disorders, called ciliopathies, the concept to which some participating medical students were exposed for the first time, allowing them to identify the gaps in knowledge and potentially strive for better future patient diagnosis.

Dr. Artoghrul Alishbayli (Radboud University, Netherlands) followed with a session on model organisms, highlighting their essential role in biological research that require careful selection based on scientific questions, evolutionary relationships, technical feasibility, and ethical considerations (Alishbayli et al., 2023; Lao-Rodríguez et al., 2023). This was a great bridge for students to move from wet-lab/pre-clinical research related concepts towards *in silico* experiments, particularly, focusing on bioinformatics applications. Dr. Elmir Mahammadov (Berlin Institute of Medical Systems Biology, Germany) further homed in on the computational perspective in neuroscience (Tyser et al., 2021; Lubatti et al., 2022), demonstrating specific applications in neuroscience and developmental biology. Finally, Dr. Rashad Yusifov (Thermo Fisher Scientific, Germany) concluded the program with discussions on translational neuroscience and clinical trials, bridging the gap between fundamental research and its application in medicine.

Each speaker's lecture was carefully designed to build upon previous topics, ensuring a logical progression of knowledge throughout the program. This collective expertise of the presenters, spanning experimental and computational neuroscience, molecular biology, and clinical applications, enriched the learning experience of the participants.

### Participation and diversity

The event attracted a diverse group of participants, predominantly university-level students and early-career researchers specializing in biology and medicine. However, a notable minority included high school students, as well as individuals from underrepresented non-traditional backgrounds, such as first-generation students and students from rural areas. An additional notable aspect of the event was the high participation of women; 60% of the participants were female and 40% were male. Additionally, the online format ensured the inclusivity of the sessions, allowing individuals from distant regions of Azerbaijan, the capital city of Baku, and those living, or studying abroad to participate.

### Impact on early-career researchers

A key success of the program was its role in fostering professional development for early-career researchers. The school provided substantial networking opportunities, enabling students to connect with instructors and peers. Many participants remained engaged in the further events organized by the ANS and sought ongoing mentorship from instructors. As a result, several students have decided to continue studies at the Master’s and/or PhD levels in the field of neuroscience, whereby the ANS board members helped them as scientific advisors and mentored these students in their future careers. Financial support was available to students in need, with more than half of them receiving it, ensuring that economic barriers did not hinder participation. The program also contributed to professional skill-building, enhancing the participants' critical thinking abilities, and informing them about key competencies, such as collaboration, communication and leadership, which are required for succeeding in the field of neuroscience and medicine.

### Future implications and next steps

**Fig. 1.**
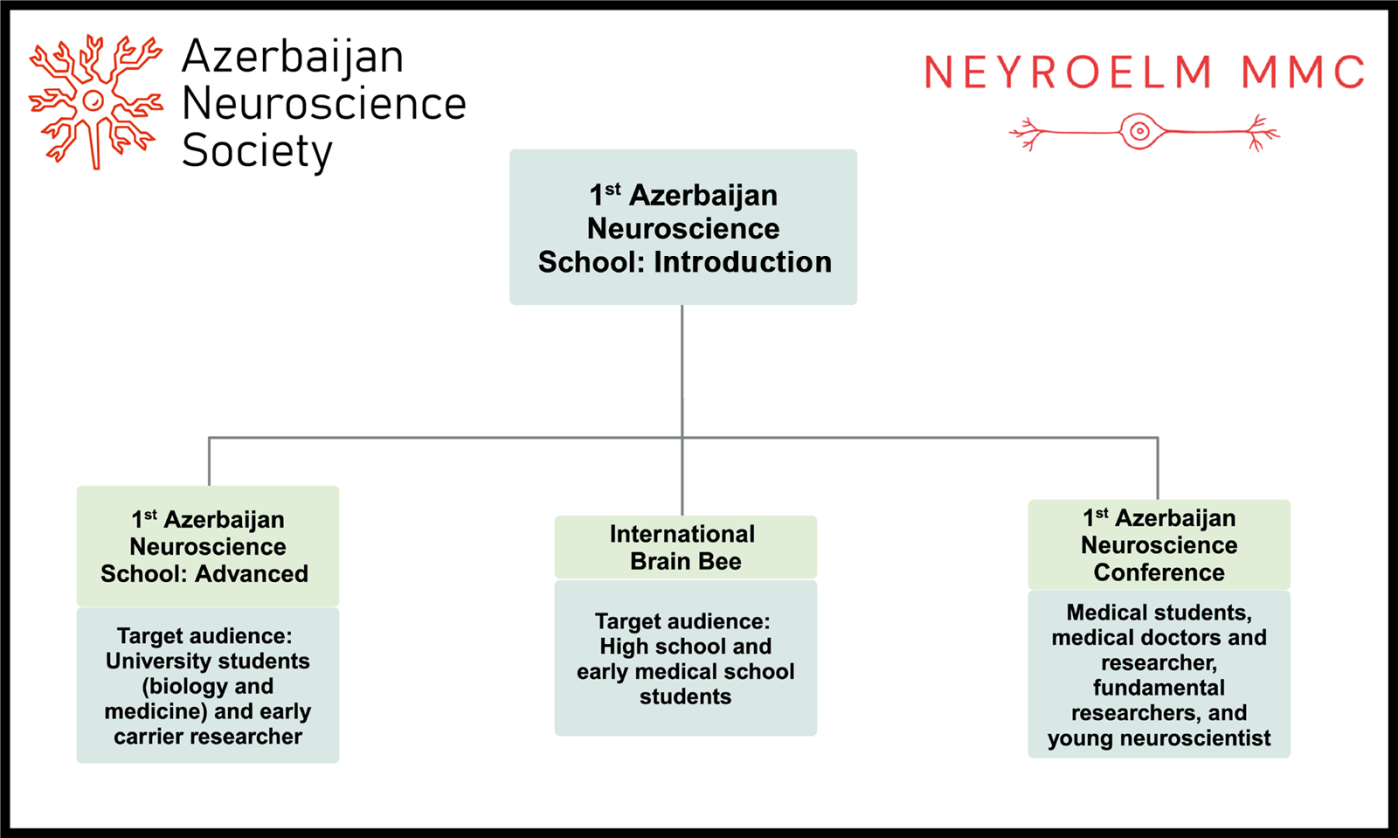
Follow-up events inspired by 1st Azerbaijani Neuroscience School: Introduction.

The knowledge and skills gained through this initiative will serve as a foundation for the participants' future careers in neuroscience and clinical fields, fostering a new generation of Azerbaijani neuroscientists. The success of the introductory school has already led to the development of an advanced-level follow-up event, the ‘Azerbaijan Neuroscience School: Advanced’ that took place from September to December 2024. The majority of students from the ‘1st Azerbaijan Neuroscience School’ participated in the follow-up ‘Advanced Neuroscience School’, as well as at the ‘1st Azerbaijan Neuroscience Conference’ on September 28th, 2024. Furthermore, attesting to the immediate impact of the school on the development of neuroscience in the country, Azerbaijan held its first ever national exam for International Brain Bee competition in July 2024, the winner of which later competed in an international competition along with the winners from other nations (Fig. 1). Collectively, these events, which were built upon the foundations laid by the introductory program, will further strengthen the neuroscience community in Azerbaijan, and will eventually solidify neuroscience as an integral part of university level education.

### Conclusion

The ‘1st Azerbaijan Neuroscience School: Introduction’ has made a lasting impact by establishing a structured neuroscience education program in Azerbaijan. By connecting students with international experts, fostering discussions on fundamental and cutting-edge topics, and creating networking opportunities, the initiative has significantly contributed to establishing neuroscience education and research in the region. With the upcoming neuroscience educational programs, including the ‘2nd Azerbaijan Neuroscience School: Introduction’, on the horizon, the future of neuroscience education in Azerbaijan looks promising, paving the way for further scientific growth and collaboration.

## References

[BIO061980C1] Alishbayli, A., Schlegel, N. J. and Englitz, B. (2023). Using auditory texture statistics for domain-neutral removal of background sounds. *Front. Audiol. Otol.* 1, 1226946. 10.3389/fauot.2023.1226946

[BIO061980C2] Baker, K. and Beales, P. L. (2009). Making sense of cilia in disease: the human ciliopathies. *Am. J. Med. Genet.* 151C, 281-295. 10.1002/ajmg.c.30231 19876933

[BIO061980C3] Bangs, F. and Anderson, K. V. (2017). Primary Cilia and Mammalian Hedgehog signaling. *Cold Spring Harb. Perspect. Biol.* 9, a028175. 10.1101/cshperspect.a028175 27881449 PMC5411695

[BIO061980C4] Lao-Rodríguez, A. B., Przewrocki, K., Pérez-González, D., Alishbayli, A., Yilmaz, E., Malmierca, M. S. and Englitz, B. (2023). Neuronal responses to omitted tones in the auditory brain: a neuronal correlate for predictive coding. *Sci. Adv.* 9, eabq8657. 10.1126/sciadv.abq8657 37315139 PMC10266733

[BIO061980C5] Lubatti, G., Mahammadov, E. and Scialdone, A. (2022). MitoHEAR: an R package for the estimation anddownstream statistical analysis of the mitochondrial DNA heteroplasmycalculated from single-cell datasets. *J. Open Source Softw.* 7, 4265. 10.21105/joss.04265

[BIO061980C6] Tyser, R. C. V., Mahammadov, E., Nakanoh, S., Vallier, L., Scialdone, A. and Srinivas, S. (2021). Single-cell transcriptomic characterization of a gastrulating human embryo. *Nature* 600, 285-289. 10.1038/s41586-021-04158-y 34789876 PMC7615353

[BIO061980C7] Yusifov, E., Dumoulin, A. and Stoeckli, E. T. (2021). Investigating primary cilia during peripheral nervous system formation. *Int. J. Mol. Sci.* 22, 3176. 10.3390/ijms22063176 33804711 PMC8003989

[BIO061980C8] Yusifov, E., Schaettin, M., Dumoulin, A., Bachmann-Gagescu, R. and Stoeckli, E. T. (2025). The primary cilium gene CPLANE1 is required for peripheral nervous system development. *Dev. Biol.* 519, 106-121. 10.1016/j.ydbio.2024.12.008 39694173

